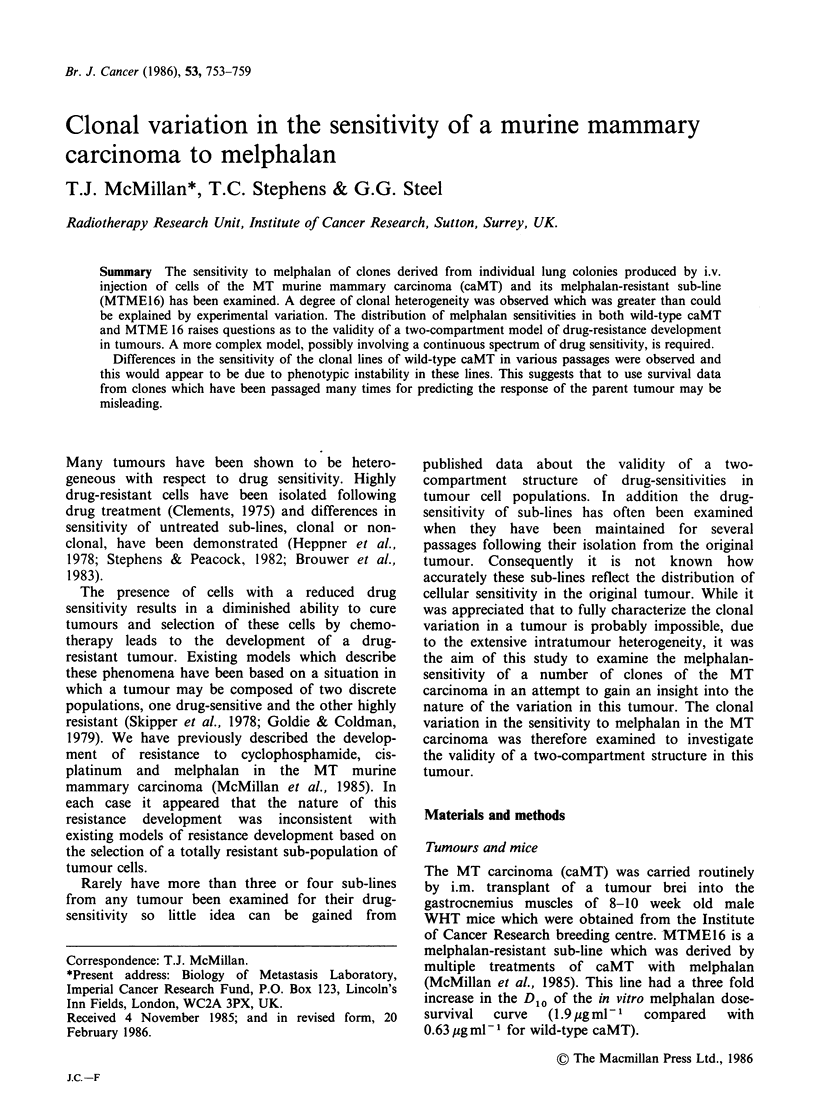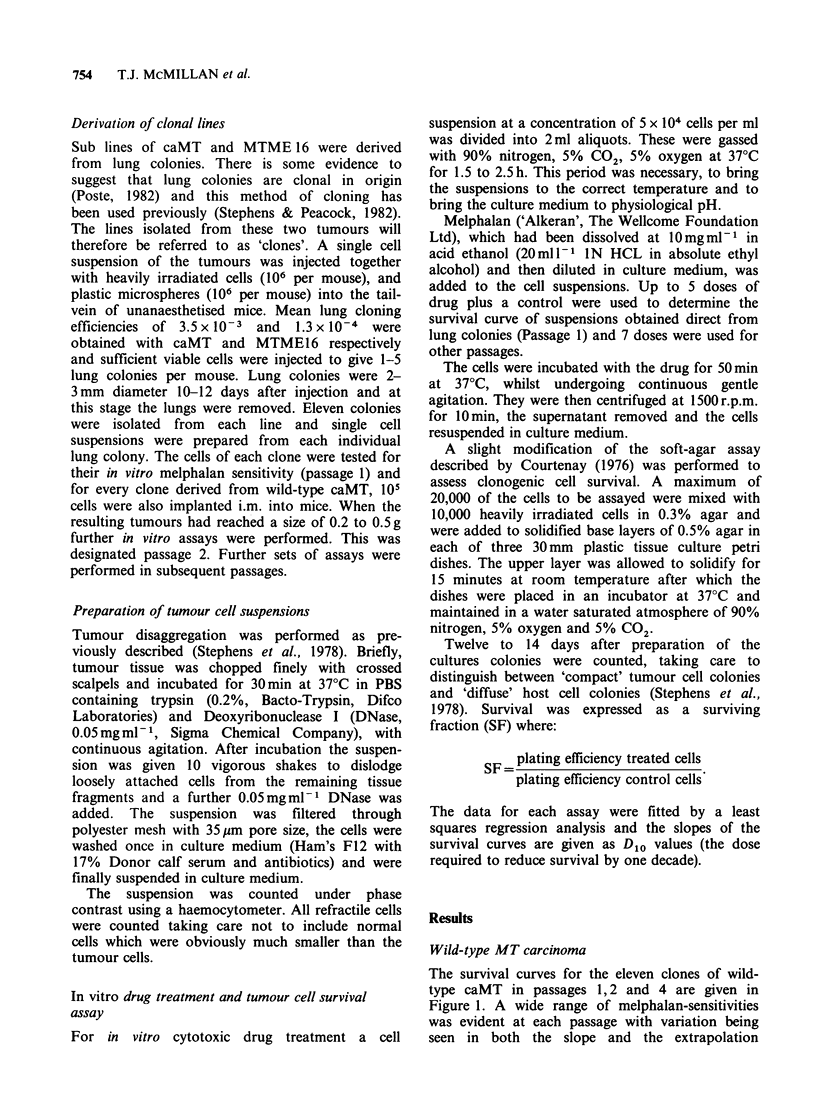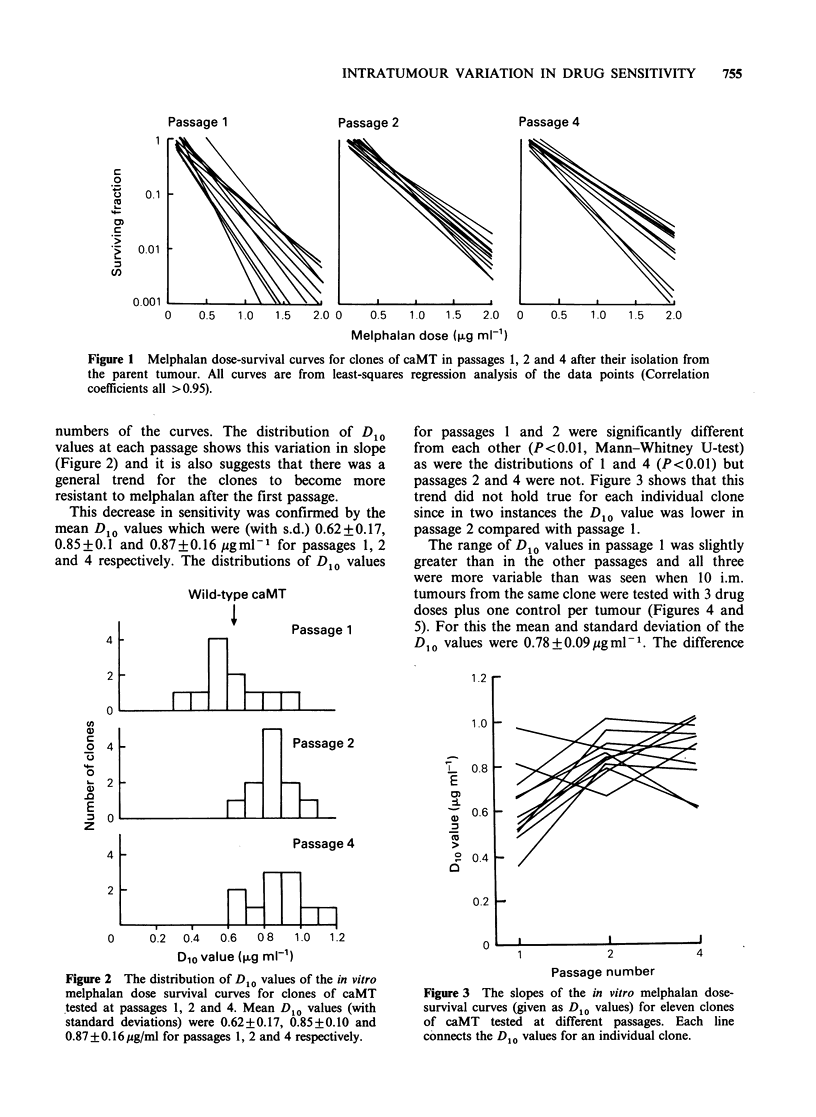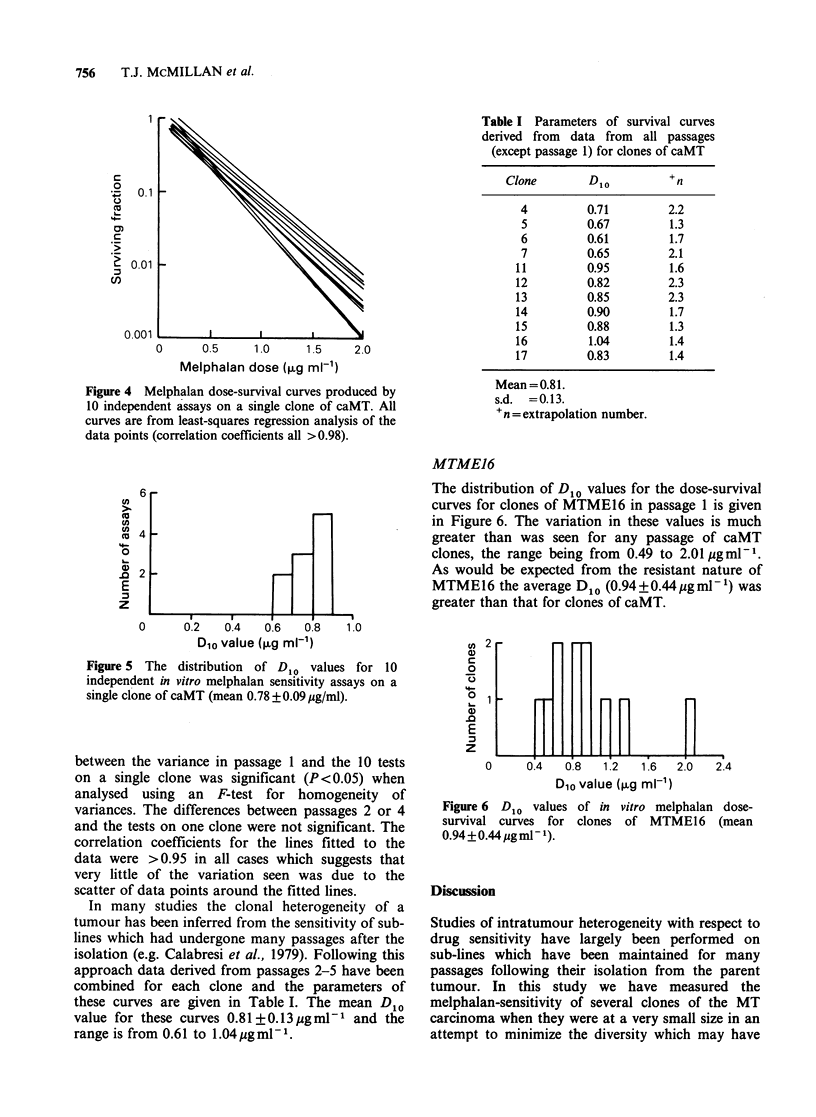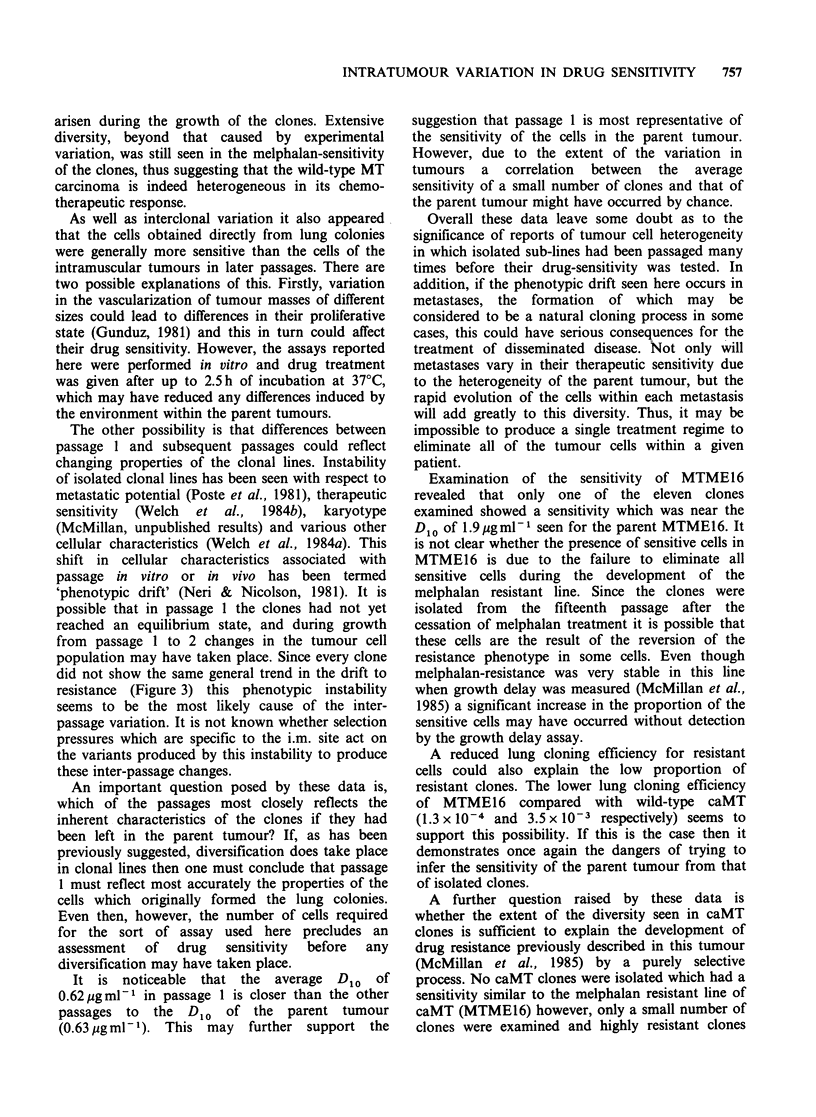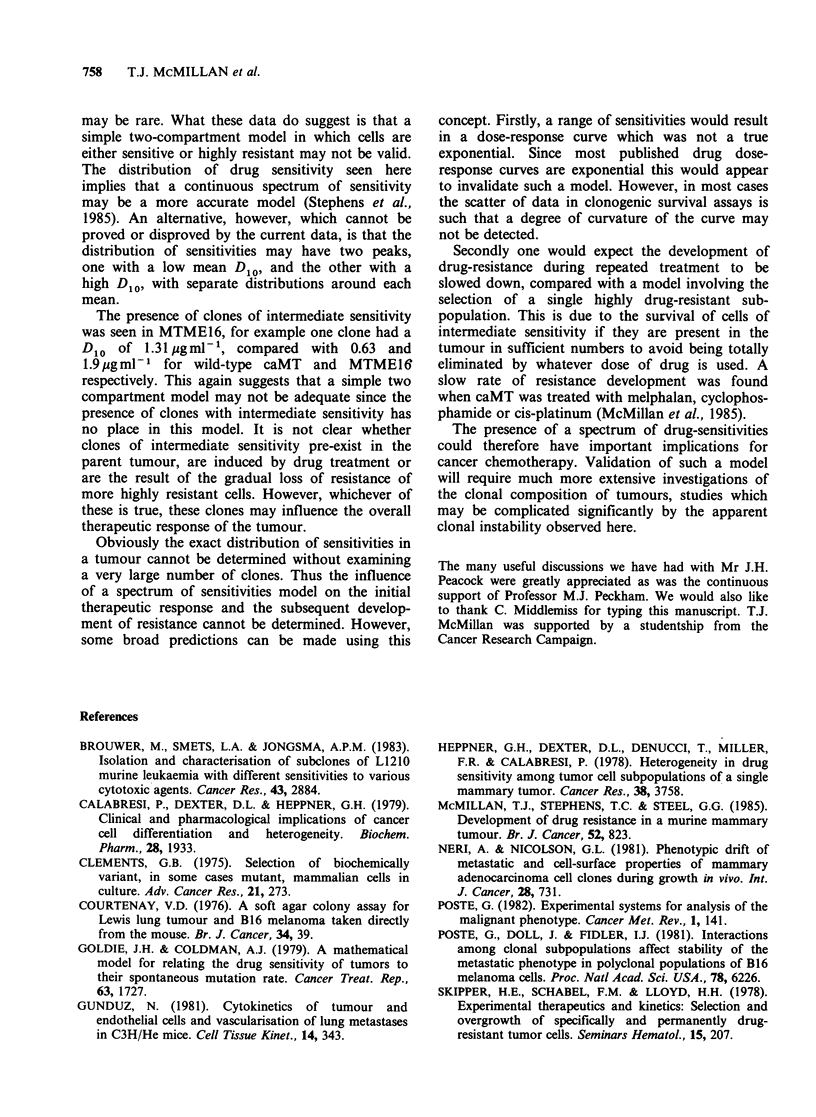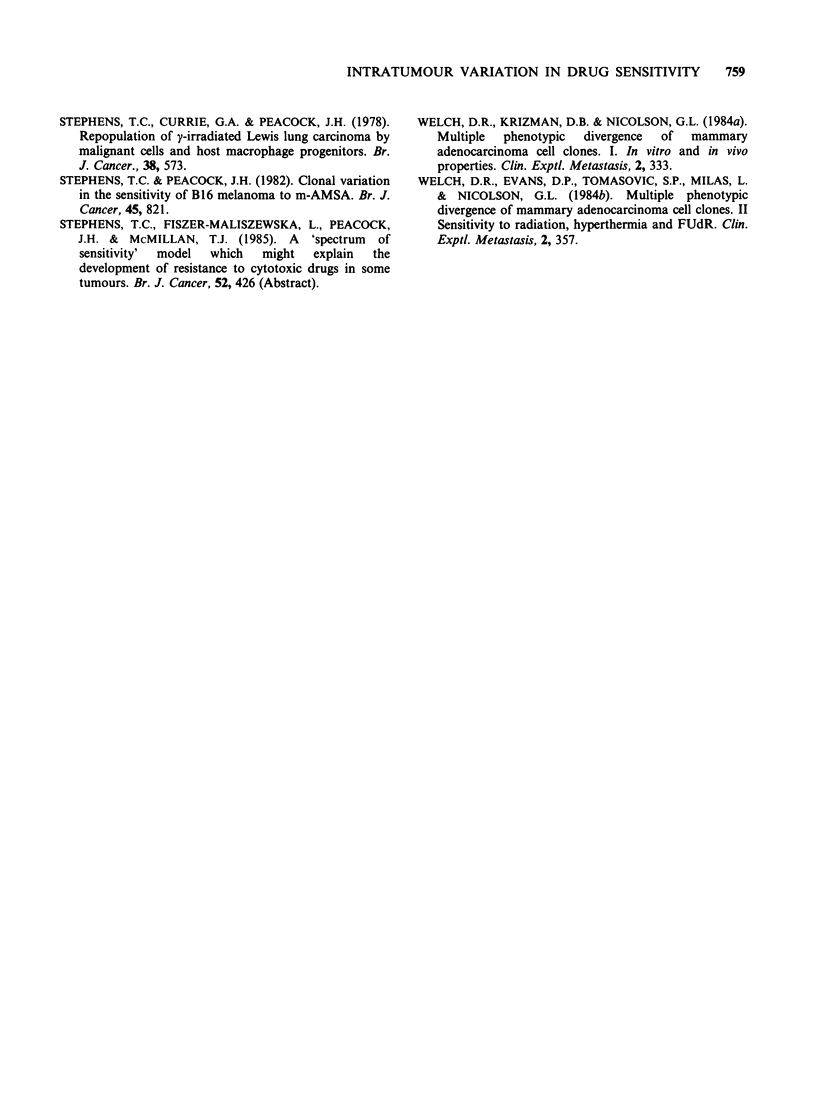# Clonal variation in the sensitivity of a murine mammary carcinoma to melphalan.

**DOI:** 10.1038/bjc.1986.129

**Published:** 1986-06

**Authors:** T. J. McMillan, T. C. Stephens, G. G. Steel

## Abstract

The sensitivity to melphalan of clones derived from individual lung colonies produced by i.v. injection of cells of the MT murine mammary carcinoma (caMT) and its melphalan-resistant sub-line (MTME16) has been examined. A degree of clonal heterogeneity was observed which was greater than could be explained by experimental variation. The distribution of melphalan sensitivities in both wild-type caMT and MTME16 raises questions as to the validity of a two-compartment model of drug-resistance development in tumours. A more complex model, possibly involving a continuous spectrum of drug sensitivity, is required. Differences in the sensitivity of the clonal lines of wild-type caMT in various passages were observed and this would appear to be due to phenotypic instability in these lines. This suggests that to use survival data from clones which have been passaged many times for predicting the response of the parent tumour may be misleading.


Br. J. Cancer (1986), 53, 753–759

### Clonal variation in the sensitivity of a murine mammary carcinoma to melphalan

T.J. McMillan*, T.C. Stephens & G.G. Steel

*Radiotherapy Research Unit, Institute of Cancer Research, Sutton, Surrey, UK.*

**Summary** The sensitivity to melphalan of clones derived from individual lung colonies produced by i.v. injection of cells of the MT murine mammary carcinoma (caMT) and its melphalan-resistant sub-line (MTME16) has been examined. A degree of clonal heterogeneity was observed which was greater than could be explained by experimental variation. The distribution of melphalan sensitivities in both wild-type caMT and MTME 16 raises questions as to the validity of a two-compartment model of drug-resistance development in tumours. A more complex model, possibly involving a continuous spectrum of drug sensitivity, is required.

Differences in the sensitivity of the clonal lines of wild-type caMT in various passages were observed and this would appear to be due to phenotypic instability in these lines. This suggests that to use survival data from clones which have been passaged many times for predicting the response of the parent tumour may be misleading.

Many tumours have been shown to be heterogeneous with respect to drug sensitivity. Highly drug-resistant cells have been isolated following drug treatment (Clements, 1975) and differences in sensitivity of untreated sub-lines, clonal or non-clonal, have been demonstrated (Heppner *et al.*, 1978; Stephens & Peacock, 1982; Brouwer *et al.*, 1983).

The presence of cells with a reduced drug sensitivity results in a diminished ability to cure tumours and selection of these cells by chemotherapy leads to the development of a drug-resistant tumour. Existing models which describe these phenomena have been based on a situation in which a tumour may be composed of two discrete populations, one drug-sensitive and the other highly resistant (Skipper *et al.*, 1978; Goldie & Coldman, 1979). We have previously described the development of resistance to cyclophosphamide, cis-platinum and melphalan in the MT murine mammary carcinoma (McMillan *et al.*, 1985). In each case it appeared that the nature of this resistance development was inconsistent with existing models of resistance development based on the selection of a totally resistant sub-population of tumour cells.

Rarely have more than three or four sub-lines from any tumour been examined for their drug-sensitivity so little idea can be gained from published data about the validity of a two-compartment structure of drug-sensitivities in tumour cell populations. In addition the drug-sensitivity of sub-lines has often been examined when they have been maintained for several passages following their isolation from the original tumour. Consequently it is not known how accurately these sub-lines reflect the distribution of cellular sensitivity in the original tumour. While it was appreciated that to fully characterize the clonal variation in a tumour is probably impossible, due to the extensive intratumour heterogeneity, it was the aim of this study to examine the melphalan-sensitivity of a number of clones of the MT carcinoma in an attempt to gain an insight into the nature of the variation in this tumour. The clonal variation in the sensitivity to melphalan in the MT carcinoma was therefore examined to investigate the validity of a two-compartment structure in this tumour.

### Materials and methods

### *Tumours and mice*

The MT carcinoma (caMT) was carried routinely by i.m. transplant of a tumour brei into the gastrocnemius muscles of 8–10 week old male WHT mice which were obtained from the Institute of Cancer Research breeding centre. MTME16 is a melphalan-resistant sub-line which was derived by multiple treatments of caMT with melphalan (McMillan *et al.*, 1985). This line had a three fold increase in the $D_{10}$ of the *in vitro* melphalan dose-survival curve (1.9 $\mu g\,ml^{-1}$ compared with 0.63 $\mu g\,ml^{-1}$ for wild-type caMT).

Correspondence: T.J. McMillan.
*Present address: Biology of Metastasis Laboratory, Imperial Cancer Research Fund, P.O. Box 123, Lincoln's Inn Fields, London, WC2A 3PX, UK.
Received 4 November 1985; and in revised form, 20 February 1986.

© The Macmillan Press Ltd., 1986

*Derivation of clonal lines*

Sub lines of caMT and MTME 16 were derived from lung colonies. There is some evidence to suggest that lung colonies are clonal in origin (Poste, 1982) and this method of cloning has been used previously (Stephens & Peacock, 1982). The lines isolated from these two tumours will therefore be referred to as 'clones'. A single cell suspension of the tumours was injected together with heavily irradiated cells ($10^6$ per mouse), and plastic microspheres ($10^6$ per mouse) into the tail-vein of unanaesthetised mice. Mean lung cloning efficiencies of $3.5\times10^{-3}$ and $1.3\times10^{-4}$ were obtained with caMT and MTME16 respectively and sufficient viable cells were injected to give 1–5 lung colonies per mouse. Lung colonies were 2–3 mm diameter 10–12 days after injection and at this stage the lungs were removed. Eleven colonies were isolated from each line and single cell suspensions were prepared from each individual lung colony. The cells of each clone were tested for their *in vitro* melphalan sensitivity (passage 1) and for every clone derived from wild-type caMT, $10^5$ cells were also implanted i.m. into mice. When the resulting tumours had reached a size of 0.2 to 0.5 g further *in vitro* assays were performed. This was designated passage 2. Further sets of assays were performed in subsequent passages.

*Preparation of tumour cell suspensions*

Tumour disaggregation was performed as previously described (Stephens *et al.*, 1978). Briefly, tumour tissue was chopped finely with crossed scalpels and incubated for 30 min at 37°C in PBS containing trypsin (0.2%, Bacto-Trypsin, Difco Laboratories) and Deoxyribonuclease I (DNase, 0.05 mg ml$^{-1}$, Sigma Chemical Company), with continuous agitation. After incubation the suspension was given 10 vigorous shakes to dislodge loosely attached cells from the remaining tissue fragments and a further 0.05 mg ml$^{-1}$ DNase was added. The suspension was filtered through polyester mesh with 35 $\mu$m pore size, the cells were washed once in culture medium (Ham's F12 with 17% Donor calf serum and antibiotics) and were finally suspended in culture medium.

The suspension was counted under phase contrast using a haemocytometer. All refractile cells were counted taking care not to include normal cells which were obviously much smaller than the tumour cells.

In vitro *drug treatment and tumour cell survival assay*

For *in vitro* cytotoxic drug treatment a cell suspension at a concentration of $5\times10^4$ cells per ml was divided into 2 ml aliquots. These were gassed with 90% nitrogen, 5% $CO_2$, 5% oxygen at 37°C for 1.5 to 2.5 h. This period was necessary, to bring the suspensions to the correct temperature and to bring the culture medium to physiological pH.

Melphalan ('Alkeran', The Wellcome Foundation Ltd), which had been dissolved at 10 mg ml$^{-1}$ in acid ethanol (20 ml l$^{-1}$ 1N HCL in absolute ethyl alcohol) and then diluted in culture medium, was added to the cell suspensions. Up to 5 doses of drug plus a control were used to determine the survival curve of suspensions obtained direct from lung colonies (Passage 1) and 7 doses were used for other passages.

The cells were incubated with the drug for 50 min at 37°C, whilst undergoing continuous gentle agitation. They were then centrifuged at 1500 r.p.m. for 10 min, the supernatant removed and the cells resuspended in culture medium.

A slight modification of the soft-agar assay described by Courtenay (1976) was performed to assess clonogenic cell survival. A maximum of 20,000 of the cells to be assayed were mixed with 10,000 heavily irradiated cells in 0.3% agar and were added to solidified base layers of 0.5% agar in each of three 30 mm plastic tissue culture petri dishes. The upper layer was allowed to solidify for 15 minutes at room temperature after which the dishes were placed in an incubator at 37°C and maintained in a water saturated atmosphere of 90% nitrogen, 5% oxygen and 5% $CO_2$.

Twelve to 14 days after preparation of the cultures colonies were counted, taking care to distinguish between 'compact' tumour cell colonies and 'diffuse' host cell colonies (Stephens *et al.*, 1978). Survival was expressed as a surviving fraction (SF) where:

$$SF=\frac{\text{plating efficiency treated cells}}{\text{plating efficiency control cells}}.$$

The data for each assay were fitted by a least squares regression analysis and the slopes of the survival curves are given as $D_{10}$ values (the dose required to reduce survival by one decade).

### Results

*Wild-type MT carcinoma*

The survival curves for the eleven clones of wild-type caMT in passages 1, 2 and 4 are given in Figure 1. A wide range of melphalan-sensitivities was evident at each passage with variation being seen in both the slope and the extrapolation

**Figure 1** Melphalan dose-survival curves for clones of caMT in passages 1, 2 and 4 after their isolation from the parent tumour. All curves are from least-squares regression analysis of the data points (Correlation coefficients all >0.95).

numbers of the curves. The distribution of $D_{10}$ values at each passage shows this variation in slope (Figure 2) and it is also suggests that there was a general trend for the clones to become more resistant to melphalan after the first passage.

This decrease in sensitivity was confirmed by the mean $D_{10}$ values which were (with s.d.) $0.62 \pm 0.17$, $0.85 \pm 0.1$ and $0.87 \pm 0.16\ \mu g\, ml^{-1}$ for passages 1, 2 and 4 respectively. The distributions of $D_{10}$ values for passages 1 and 2 were significantly different from each other ($P<0.01$, Mann–Whitney U-test) as were the distributions of 1 and 4 ($P<0.01$) but passages 2 and 4 were not. Figure 3 shows that this trend did not hold true for each individual clone since in two instances the $D_{10}$ value was lower in passage 2 compared with passage 1.

The range of $D_{10}$ values in passage 1 was slightly greater than in the other passages and all three were more variable than was seen when 10 i.m. tumours from the same clone were tested with 3 drug doses plus one control per tumour (Figures 4 and 5). For this the mean and standard deviation of the $D_{10}$ values were $0.78 \pm 0.09\ \mu g\, ml^{-1}$. The difference

**Figure 2** The distribution of $D_{10}$ values of the *in vitro* melphalan dose survival curves for clones of caMT tested at passages 1, 2 and 4. Mean $D_{10}$ values (with standard deviations) were $0.62 \pm 0.17$, $0.85 \pm 0.10$ and $0.87 \pm 0.16\ \mu g/ml$ for passages 1, 2 and 4 respectively.

**Figure 3** The slopes of the *in vitro* melphalan dose-survival curves (given as $D_{10}$ values) for eleven clones of caMT tested at different passages. Each line connects the $D_{10}$ values for an individual clone.

**Figure 4** Melphalan dose-survival curves produced by 10 independent assays on a single clone of caMT. All curves are from least-squares regression analysis of the data points (correlation coefficients all >0.98).

**Figure 5** The distribution of $D_{10}$ values for 10 independent *in vitro* melphalan sensitivity assays on a single clone of caMT (mean $0.78 \pm 0.09$ μg/ml).

between the variance in passage 1 and the 10 tests on a single clone was significant ($P<0.05$) when analysed using an $F$-test for homogeneity of variances. The differences between passages 2 or 4 and the tests on one clone were not significant. The correlation coefficients for the lines fitted to the data were >0.95 in all cases which suggests that very little of the variation seen was due to the scatter of data points around the fitted lines.

In many studies the clonal heterogeneity of a tumour has been inferred from the sensitivity of sub-lines which had undergone many passages after the isolation (e.g. Calabresi *et al.*, 1979). Following this approach data derived from passages 2–5 have been combined for each clone and the parameters of these curves are given in Table I. The mean $D_{10}$ value for these curves $0.81 \pm 0.13$ μg ml$^{-1}$ and the range is from 0.61 to 1.04 μg ml$^{-1}$.

**Table I** Parameters of survival curves derived from data from all passages (except passage 1) for clones of caMT

| *Clone* | $D_{10}$ | $^{+}n$ |
|---|---|---|
| 4 | 0.71 | 2.2 |
| 5 | 0.67 | 1.3 |
| 6 | 0.61 | 1.7 |
| 7 | 0.65 | 2.1 |
| 11 | 0.95 | 1.6 |
| 12 | 0.82 | 2.3 |
| 13 | 0.85 | 2.3 |
| 14 | 0.90 | 1.7 |
| 15 | 0.88 | 1.3 |
| 16 | 1.04 | 1.4 |
| 17 | 0.83 | 1.4 |

Mean = 0.81.
s.d. = 0.13.
$^{+}n$ = extrapolation number.

### *MTME16*

The distribution of $D_{10}$ values for the dose-survival curves for clones of MTME16 in passage 1 is given in Figure 6. The variation in these values is much greater than was seen for any passage of caMT clones, the range being from 0.49 to 2.01 μg ml$^{-1}$. As would be expected from the resistant nature of MTME16 the average $D_{10}$ ($0.94 \pm 0.44$ μg ml$^{-1}$) was greater than that for clones of caMT.

**Figure 6** $D_{10}$ values of *in vitro* melphalan dose-survival curves for clones of MTME16 (mean $0.94 \pm 0.44$ μg ml$^{-1}$).

### Discussion

Studies of intratumour heterogeneity with respect to drug sensitivity have largely been performed on sub-lines which have been maintained for many passages following their isolation from the parent tumour. In this study we have measured the melphalan-sensitivity of several clones of the MT carcinoma when they were at a very small size in an attempt to minimize the diversity which may have arisen during the growth of the clones. Extensive diversity, beyond that caused by experimental variation, was still seen in the melphalan-sensitivity of the clones, thus suggesting that the wild-type MT carcinoma is indeed heterogeneous in its chemotherapeutic response.

As well as interclonal variation it also appeared that the cells obtained directly from lung colonies were generally more sensitive than the cells of the intramuscular tumours in later passages. There are two possible explanations of this. Firstly, variation in the vascularization of tumour masses of different sizes could lead to differences in their proliferative state (Gunduz, 1981) and this in turn could affect their drug sensitivity. However, the assays reported here were performed *in vitro* and drug treatment was given after up to 2.5 h of incubation at 37°C, which may have reduced any differences induced by the environment within the parent tumours.

The other possibility is that differences between passage 1 and subsequent passages could reflect changing properties of the clonal lines. Instability of isolated clonal lines has been seen with respect to metastatic potential (Poste *et al.*, 1981), therapeutic sensitivity (Welch *et al.*, 1984*b*), karyotype (McMillan, unpublished results) and various other cellular characteristics (Welch *et al.*, 1984*a*). This shift in cellular characteristics associated with passage *in vitro* or *in vivo* has been termed 'phenotypic drift' (Neri & Nicolson, 1981). It is possible that in passage 1 the clones had not yet reached an equilibrium state, and during growth from passage 1 to 2 changes in the tumour cell population may have taken place. Since every clone did not show the same general trend in the drift to resistance (Figure 3) this phenotypic instability seems to be the most likely cause of the inter-passage variation. It is not known whether selection pressures which are specific to the i.m. site act on the variants produced by this instability to produce these inter-passage changes.

An important question posed by these data is, which of the passages most closely reflects the inherent characteristics of the clones if they had been left in the parent tumour? If, as has been previously suggested, diversification does take place in clonal lines then one must conclude that passage 1 must reflect most accurately the properties of the cells which originally formed the lung colonies. Even then, however, the number of cells required for the sort of assay used here precludes an assessment of drug sensitivity before any diversification may have taken place.

It is noticeable that the average $D_{10}$ of $0.62\,\mu g\,ml^{-1}$ in passage 1 is closer than the other passages to the $D_{10}$ of the parent tumour ($0.63\,\mu g\,ml^{-1}$). This may further support the suggestion that passage 1 is most representative of the sensitivity of the cells in the parent tumour. However, due to the extent of the variation in tumours a correlation between the average sensitivity of a small number of clones and that of the parent tumour might have occurred by chance.

Overall these data leave some doubt as to the significance of reports of tumour cell heterogeneity in which isolated sub-lines had been passaged many times before their drug-sensitivity was tested. In addition, if the phenotypic drift seen here occurs in metastases, the formation of which may be considered to be a natural cloning process in some cases, this could have serious consequences for the treatment of disseminated disease. Not only will metastases vary in their therapeutic sensitivity due to the heterogeneity of the parent tumour, but the rapid evolution of the cells within each metastasis will add greatly to this diversity. Thus, it may be impossible to produce a single treatment regime to eliminate all of the tumour cells within a given patient.

Examination of the sensitivity of MTME16 revealed that only one of the eleven clones examined showed a sensitivity which was near the $D_{10}$ of $1.9\,\mu g\,ml^{-1}$ seen for the parent MTME16. It is not clear whether the presence of sensitive cells in MTME16 is due to the failure to eliminate all sensitive cells during the development of the melphalan resistant line. Since the clones were isolated from the fifteenth passage after the cessation of melphalan treatment it is possible that these cells are the result of the reversion of the resistance phenotype in some cells. Even though melphalan-resistance was very stable in this line when growth delay was measured (McMillan *et al.*, 1985) a significant increase in the proportion of the sensitive cells may have occurred without detection by the growth delay assay.

A reduced lung cloning efficiency for resistant cells could also explain the low proportion of resistant clones. The lower lung cloning efficiency of MTME16 compared with wild-type caMT ($1.3\times10^{-4}$ and $3.5\times10^{-3}$ respectively) seems to support this possibility. If this is the case then it demonstrates once again the dangers of trying to infer the sensitivity of the parent tumour from that of isolated clones.

A further question raised by these data is whether the extent of the diversity seen in caMT clones is sufficient to explain the development of drug resistance previously described in this tumour (McMillan *et al.*, 1985) by a purely selective process. No caMT clones were isolated which had a sensitivity similar to the melphalan resistant line of caMT (MTME16) however, only a small number of clones were examined and highly resistant clones may be rare. What these data do suggest is that a simple two-compartment model in which cells are either sensitive or highly resistant may not be valid. The distribution of drug sensitivity seen here implies that a continuous spectrum of sensitivity may be a more accurate model (Stephens *et al.*, 1985). An alternative, however, which cannot be proved or disproved by the current data, is that the distribution of sensitivities may have two peaks, one with a low mean $D_{10}$, and the other with a high $D_{10}$, with separate distributions around each mean.

The presence of clones of intermediate sensitivity was seen in MTME16, for example one clone had a $D_{10}$ of 1.31 $\mu g\,ml^{-1}$, compared with 0.63 and 1.9 $\mu g\,ml^{-1}$ for wild-type caMT and MTME16 respectively. This again suggests that a simple two compartment model may not be adequate since the presence of clones with intermediate sensitivity has no place in this model. It is not clear whether clones of intermediate sensitivity pre-exist in the parent tumour, are induced by drug treatment or are the result of the gradual loss of resistance of more highly resistant cells. However, whichever of these is true, these clones may influence the overall therapeutic response of the tumour.

Obviously the exact distribution of sensitivities in a tumour cannot be determined without examining a very large number of clones. Thus the influence of a spectrum of sensitivities model on the initial therapeutic response and the subsequent development of resistance cannot be determined. However, some broad predictions can be made using this concept. Firstly, a range of sensitivities would result in a dose-response curve which was not a true exponential. Since most published drug dose-response curves are exponential this would appear to invalidate such a model. However, in most cases the scatter of data in clonogenic survival assays is such that a degree of curvature of the curve may not be detected.

Secondly one would expect the development of drug-resistance during repeated treatment to be slowed down, compared with a model involving the selection of a single highly drug-resistant sub-population. This is due to the survival of cells of intermediate sensitivity if they are present in the tumour in sufficient numbers to avoid being totally eliminated by whatever dose of drug is used. A slow rate of resistance development was found when caMT was treated with melphalan, cyclophosphamide or cis-platinum (McMillan *et al.*, 1985).

The presence of a spectrum of drug-sensitivities could therefore have important implications for cancer chemotherapy. Validation of such a model will require much more extensive investigations of the clonal composition of tumours, studies which may be complicated significantly by the apparent clonal instability observed here.

The many useful discussions we have had with Mr J.H. Peacock were greatly appreciated as was the continuous support of Professor M.J. Peckham. We would also like to thank C. Middlemiss for typing this manuscript. T.J. McMillan was supported by a studentship from the Cancer Research Campaign.

**References**

BROUWER, M., SMETS, L.A. & JONGSMA, A.P.M. (1983). Isolation and characterisation of subclones of L1210 murine leukaemia with different sensitivities to various cytotoxic agents. *Cancer Res.*, **43**, 2884.

CALABRESI, P., DEXTER, D.L. & HEPPNER, G.H. (1979). Clinical and pharmacological implications of cancer cell differentiation and heterogeneity. *Biochem. Pharm.*, **28**, 1933.

CLEMENTS, G.B. (1975). Selection of biochemically variant, in some cases mutant, mammalian cells in culture. *Adv. Cancer Res.*, **21**, 273.

COURTENAY, V.D. (1976). A soft agar colony assay for Lewis lung tumour and B16 melanoma taken directly from the mouse. *Br. J. Cancer*, **34**, 39.

GOLDIE, J.H. & COLDMAN, A.J. (1979). A mathematical model for relating the drug sensitivity of tumors to their spontaneous mutation rate. *Cancer Treat. Rep.*, **63**, 1727.

GUNDUZ, N. (1981). Cytokinetics of tumour and endothelial cells and vascularisation of lung metastases in C3H/He mice. *Cell Tissue Kinet.*, **14**, 343.

HEPPNER, G.H., DEXTER, D.L., DENUCCI, T., MILLER, F.R. & CALABRESI, P. (1978). Heterogeneity in drug sensitivity among tumor cell subpopulations of a single mammary tumor. *Cancer Res.*, **38**, 3758.

McMILLAN, T.J., STEPHENS, T.C. & STEEL, G.G. (1985). Development of drug resistance in a murine mammary tumour. *Br. J. Cancer*, **52**, 823.

NERI, A. & NICOLSON, G.L. (1981). Phenotypic drift of metastatic and cell-surface properties of mammary adenocarcinoma cell clones during growth *in vivo*. *Int. J. Cancer*, **28**, 731.

POSTE, G. (1982). Experimental systems for analysis of the malignant phenotype. *Cancer Met. Rev.*, **1**, 141.

POSTE, G., DOLL, J. & FIDLER, I.J. (1981). Interactions among clonal subpopulations affect stability of the metastatic phenotype in polyclonal populations of B16 melanoma cells. *Proc. Natl Acad. Sci. USA.*, **78**, 6226.

SKIPPER, H.E., SCHABEL, F.M. & LLOYD, H.H. (1978). Experimental therapeutics and kinetics: Selection and overgrowth of specifically and permanently drug-resistant tumor cells. *Seminars Hematol.*, **15**, 207.

STEPHENS, T.C., CURRIE, G.A. & PEACOCK, J.H. (1978). Repopulation of $\gamma$-irradiated Lewis lung carcinoma by malignant cells and host macrophage progenitors. *Br. J. Cancer.*, **38**, 573.

STEPHENS, T.C. & PEACOCK, J.H. (1982). Clonal variation in the sensitivity of B16 melanoma to m-AMSA. *Br. J. Cancer*, **45**, 821.

STEPHENS, T.C., FISZER-MALISZEWSKA, L., PEACOCK, J.H. & MCMILLAN, T.J. (1985). A 'spectrum of sensitivity' model which might explain the development of resistance to cytotoxic drugs in some tumours. *Br. J. Cancer*, **52**, 426 (Abstract).

WELCH, D.R., KRIZMAN, D.B. & NICOLSON, G.L. (1984*a*). Multiple phenotypic divergence of mammary adenocarcinoma cell clones. I. *In vitro* and *in vivo* properties. *Clin. Exptl. Metastasis*, **2**, 333.

WELCH, D.R., EVANS, D.P., TOMASOVIC, S.P., MILAS, L. & NICOLSON, G.L. (1984*b*). Multiple phenotypic divergence of mammary adenocarcinoma cell clones. II Sensitivity to radiation, hyperthermia and FUdR. *Clin. Exptl. Metastasis*, **2**, 357.